# Imprinting aberrations of *SNRPN*, *ZAC1* and *INPP5F* genes involved in the pathogenesis of congenital heart disease with extracardiac malformations

**DOI:** 10.1111/jcmm.15584

**Published:** 2020-07-21

**Authors:** Xiaolei Zhao, Shaoyan Chang, Xinli Liu, Shuangxing Wang, Yueran Zhang, Xiaolin Lu, Ting Zhang, Hui Zhang, Li Wang

**Affiliations:** ^1^ Department of Cardiac Surgery The Capital Institute of Pediatrics Beijing China; ^2^ Municipal Key Laboratory of Child Development and Nutriomics Capital Institute of Pediatrics Beijing China; ^3^ Department of Obstetrics and Gynecology PLA Army General Hospital 263rd Clinical Department Beijing China

**Keywords:** CHD with EM, imprinted genes, methylation

## Abstract

Congenital heart disease (CHD) with extracardiac malformations (EM) is the most common multiple malformation, resulting from the interaction between genetic abnormalities and environmental factors. Most studies have attributed the causes of CHD with EM to chromosomal abnormalities. However, multi‐system dysplasia is usually caused by both genetic mutations and epigenetic dysregulation. The epigenetic mechanisms underlying the pathogenesis of CHD with EM remain unclear. In this study, we investigated the mechanisms of imprinting alterations, including those of the Small nuclear ribonucleoprotein polypeptide N (*SNRPN*), PLAG1 like zinc finger 1 (*ZAC1*) and inositol polyphosphate‐5‐phosphatase F (*INPP5F*) genes, in the pathogenesis of CHD with EM. The methylation levels of *SNRPN*, *ZAC1,* and *INPP5F* genes were analysed by the MassARRAY platform in 24 children with CHD with EM and 20 healthy controls. The expression levels of these genes were detected by real‐time polymerase chain reaction (PCR). The correlation between methylation regulation and gene expression was confirmed using 5‐azacytidine (5‐Aza) treated cells. The methylation levels of *SNRPN* and *ZAC1* genes were significantly increased in CHD with EM, while that of *INPP5F* was decreased. The methylation alterations of these genes were negatively correlated with expression. Risk analysis showed that abnormal hypermethylation of *SNRPN* and *ZAC1* resulted in 5.545 and 7.438 times higher risks of CHD with EM, respectively, and the abnormal hypomethylation of *INPP5F* was 8.38 times higher than that of the control group. We concluded that abnormally high methylation levels of *SNRPN* and *ZAC1* and decreased levels of *INPP5F* imply an increased risk of CHD with EM by altering their gene functions. This study provides evidence of imprinted regulation in the pathogenesis of multiple malformations.

## 1 INTRODUCTION

Many types of congenital malformations, such as congenital heart disease (CHD), digestive system malformation, and urinary system malformation, have a greater impact on morbidity and mortality in children than that of single deformity.^1^ Studies have shown that 7%–50% of foetuses with CHD are accompanied by extracardiac malformations (EM), especially in patients with a ventricular septal defect (VSD) and right ventricular double outlet (DORV).^2^ Malformations in the urinary system, gastrointestinal system, and nervous system are the most common external deformities, while malformations in the respiratory system and skeletal system are relatively rare.^2^, ^3^, ^4^, ^5^ EM significantly interrupt the natural history and clinical course of CHD.^1^ Due to the mutual influence of CHD with EM, the treatment fee is expensive, but the prognosis is unsatisfactory. Therefore, it is important to understand the pathogenesis of multiple malformations in CHD with EM.

Most studies have attributed the aetiology of syndromes containing CHD with EM to the chromosomal abnormalities and copy number variations (CNVs), such as Down syndrome and Edwards syndrome. However, the aetiology of CHD with EM is not only related to genetic variation, but also epigenetics. Environmental factors are essential for the occurrence of multi‐system dysplasia because they can facilitate the generation of epigenetic information related to phenotypic variation and disease,^6^ ultimately leading to increased susceptibility to congenital diseases in offspring.^7^ Adverse environments such as maternal diseases, nutrition, and even the atmosphere during embryonic development could disturb epigenetic modifications, leading to dysplasia or embryonic lethality.^8^ Chamberlain et al 2014 reported that environmental factors during early embryonic development could lead to CHD by influencing the methylation level of heart development‐related genes.^9^ Previous studies have suggested that smoking factors could change the methylation level of the *ERCC1* and *ADP‐ribose* genes in embryos. Additionally, ethanol could lead to the abnormal methylation of *PTNP11* or *WBSCR1* and *WBSCR22* genes, which causes Noonan or Williams syndrome.^10^ Zhou et al 2018 reported that arsenic exposure could affect the *LINE1* and *P16* methylation levels, producing genome‐wide methylation abnormalities, as well as CHD and other diseases.^11^ However, methylation alterations are effective factors of chromosome structure stability. Mollar et al 2019 reported that methylation modifications occurring during all periods of mitosis could affect kinetochore and chromosome condensation and segregation, which are essential for genome stability.^12^ Thus, abnormal methylation modification reduces the integral stability of chromosomes, inducing a ‘metastable’ status of these genomes that could result in chromosomal deletion and abnormal replication.^13^

Imprinted genes comprise elements within the human chromosome that are controlled by epigenetic modifications during early embryonic development.^14^ Appropriate imprinting levels of these genes play an essential role in embryonic development,^15^, ^16^, ^17^ and genome imprinting substantially affects the development and function of body systems, especially in embryonic stage.^8^ Changes in the imprinting level can affect the susceptibility and immunity of certain diseases.^18^ Changes in methylation modification can alter the traditional genetic balance of parents, finally affecting the development process of embryos, manifesting as multi‐system dysplasia.^19^ Presently, the mechanism underlying DNA methylation of imprinted genes in CHD with EM remains unclear. Small nuclear ribonucleoprotein polypeptide N (*SNRPN*), PLAG1 like zinc finger 1 (*ZAC1*) and inositol polyphosphate‐5‐phosphatase F (*INPP5F*) are imprinted genes that play essential roles in embryogenesis, especially cardiac development. *SNRPN* plays a vital role in specific tissues and organ development, including the heart and brain. *ZAC1* is an important transcription factor in heart development and influences heart formation. *INPP5F* is highly expressed in the heart, brain, and other tissues.

In the present study, we selected three imprinted genes, *SNRPN*, *ZAC1*, and *INPP5F*, to study the possible role of DNA methylation modifications in the aetiology of CHD with EM. We focused on children with CHD with EM and analysed the methylation levels of germinal different methylation regions (gDMRs) of the selected three imprinted genes to explore the roles of imprinting establishment on CHD with EM. This study provides a theoretical basis for the pathogenesis of CHD with EM.

## 2 MATERIALS AND METHODS

### 2.1 Sample collection

Total of forty‐four samples were collected from December 2015 to January 2018 obtained from patients treated in the Capital Institute of Pediatrics in Beijing, China, including 20 controls from children undergoing a health checkup in the Growth and Development Department and 24 CHD with EM patients from Cardiac Surgery and Medical Care Department. 24 cases were diagnosed as multiple malformations by using ultrasonic cardiograph, computed tomography (CT), X‐ray, and B‐ultrasound according to the International Classification of Disease, Tenth Revision. These patients included 10 females and 14 males with aged of 2m‐9y  , especially. Detailed information including types of CHDs and EM and corresponding sample numbers were shown in Table 1. In general, including nine cases with urinary system diseases (cryptorchidism and), eight cases with digestive system diseases (indirect inguinal hernia, imperforate anus, and abdominal hernia), four cases with nervous system diseases (terminal filament and spina bifida), three cases with motor system diseases (polydactylism, ganglion cysts, and cleft palate) and four cases with other system diseases (hemangioma and sebaceous adenoma). Control samples were confirmed to be disease‐free, including 8 females and 12 males with aged of 6m‐6y. Blood samples with detailed clinical information were collected by local trained doctors. All participants provided their informed consent, and the study protocol was reviewed and approved by the Institutional Review Board of the Capital Institute of Pediatrics.

**Table 1:** Characteristics of cases with CHD with EM and control subjects

Type of CHDs and EM		Number of cases		Age(months) Mean（min‐max）
		Male	Female	
CHDs^a^	VSD	6	5	19 (2‐108)
	ASD	5	6	15 (2‐72)
	PDA	0	4	2.3 (2‐3)
	COA	2	2	30 (2‐108)
	TOF	1	0	8
	PS	1	1	6
	PAS	1	0	5
	PLSVC	0	2	2
	others	1	1	8 (4‐12)
EM^b^	Urinary system	8	1	23 (6‐108)
	Digestive system	5	3	17 (2‐72)
	Nervous system	2	2	5 (2‐8)
	Motor system	0	3	15 (2‐36)
	others	0	4	9 (2‐16)
Total cases		14	10	18 (2‐108)
Normal controls		12	8	32 (6‐72)

^a^ VSD: ventricular septal defect; ASD: atrial septal defect; PDA: patent ductus arteriosus; COA: coarctation of the aorta; TOF: tetralogy of Fallot; PS: pulmonary stenosis; PAS: pulmonary artery sling; PLSVC: persistent left superior vena cava.

^b^ EM: extracardiac malformations in CHD cases, including the following: 1) urinary system diseases: cryptorchidism,; 2) digestive system diseases: indirect inguinal hernia, imperforate anus, abdominal hernia; 3) nervous system diseases:, spina bifida; 4) motor system diseases:, ganglion cysts, cleft palate; 5) others: hemangioma, sebaceous adenoma.

### 2.2 Cell culture

The human colorectal cancer HCT15 cell line, which demonstrates global hypermethylation, was obtained from the American Type Culture Collection . The cells were cultured at 37°C in a humidified 5% CO_2_ atmosphere in RPMI 1640 medium (Invitrogen) supplemented with 10% foetal bovine serum (GIBCO). Cells in the exponential growth phase were used for subsequent experiments. For demethylation studies, cultured cells were incubated for 72 h in 0 or 50μmol/l of 5‐azacytidine (5‐Aza; Sigma‐Aldrich), a methylation inhibitor, and the medium was changed daily.

### 2.3 DNA extraction

Genomic DNA was extracted from 200 μl of blood from human pathological samples using the Blood Genomic DNA Mini Kit (CW2087; CWBIO, Taizhou) according to the manufacturer's instructions. Genomic DNA from cells was extracted using the DNeasy® Blood & Tissue Kit (QIAGEN) according to the manufacturer's instructions. DNA with an OD260/OD280 absorbance ratio of 1.8‐1.9 was used for subsequent analysis.

### 2.4 Bisulphite treatment

In total, 500 ng of genomic DNA from each brain tissue sample was subjected to bisulphite treatment using the EZ DNA methylation kit (Zymo Research) according to the manufacturer's instructions.

### 2.5 Methylation analysis of imprinted genes

The Sequenom MassARRAY platform (CapitalBio) was used to perform quantitative analysis of gene methylation. This system uses matrix‐assisted laser desorption/ionization time‐of‐flight (MALDI‐TOF) mass spectrometry combined with RNA base‐specific cleavage (MassCLEAVE). The detected pattern is then analysed for its methylation status. Polymerase chain reaction (PCR) primers were designed using Meth primer (http://epidesigner.com). For each reverse primer, an additional T7 promoter tag for in vivo transcription was added, as well as a 10‐mer tag on the forward primer to adjust for melting temperature differences. One pair of primers was used to amplify the promoter region of each gene. The list of primers is shown in Supplementary S1. Primers were synthesized by Sangon Biotech (Shanghai, China). The spectra methylation ratios were generated by Epityper software version 1.0 (Sequenom).

### 2.6 RNA extraction and reverse transcription

RNA Extraction was performed using the RNeasy® Mini Kit (DP443; QIAGEN). For real‐time PCR, 1μg of total RNA was reverse transcribed into cDNA using the Protoscript® First Strand cDNA Synthesis Kit (NEB) according to the manufacturer's instructions. cDNA was stored at ‐20 °C until required for use in real‐time PCR.

### 2.7 Real‐time PCR

Real‐time PCR was carried out to compare the mRNA expression levels of *SNRPN*, *ZAC1* and *INPP5F* relative to that of the housekeeping gene *glyceraldehyde‐3‐phosphate dehydrogenase* (*GAPDH*). The primers were designed using Primer Express® software Version 3.0 (Applied Biosystems). All primers used are shown in Supplementary S1.

Real‐time PCR was performed using the 7500 Fast Real‐Time PCR system (Applied Biosystems) with the SYBR Green PCR Master Mix (Applied Biosystems). Each PCR reaction comprised 10 μL of 2 × SYBR Green Master Mix, 0.4 μL of forward primer, 0.4 μL of reverse primer, 1 μL of cDNA template, and 8.2 μL H_2_O; reactions were run in triplicate. The thermal cycling conditions were as follows: 50°C for 20 s, 95°C for 10 min, and then 40 cycles of 95°C for 15 s and 60°C for 1 min. The expression levels of the target genes were calculated using the 2^‐ΔΔCT^ method, for which ΔCT = CT_target gene_–CT_gapdh_.

### 2.8 Statistical analyses

The data were stored in the EPI 3.1 Database (EpiData Association) and were analysed using the SPSS 18.0 software package (McGraw‐Hill Inc). The methylation level of the imprinted genes was compared between CHD with EM and the control groups by independent sample t test. One‐way analysis of variance (ANOVA) was performed to evaluate the differences among different EM systems and CHD subgroups. Odds ratios (ORs) were calculated to evaluate the incidence of CHD with EM correlating with the methylation levels. The data were shown as means and standard deviation. All *p*‐values were two‐sided, and *P* < .05 was considered significant. GraphPad Prism 7 software (GraphPad Software) was used to display the analysis results visually.

## 3 RESULTS

### 3.1 Abnormal methylation modifications of imprinted genes in CHD with EM

**Figure 1:**
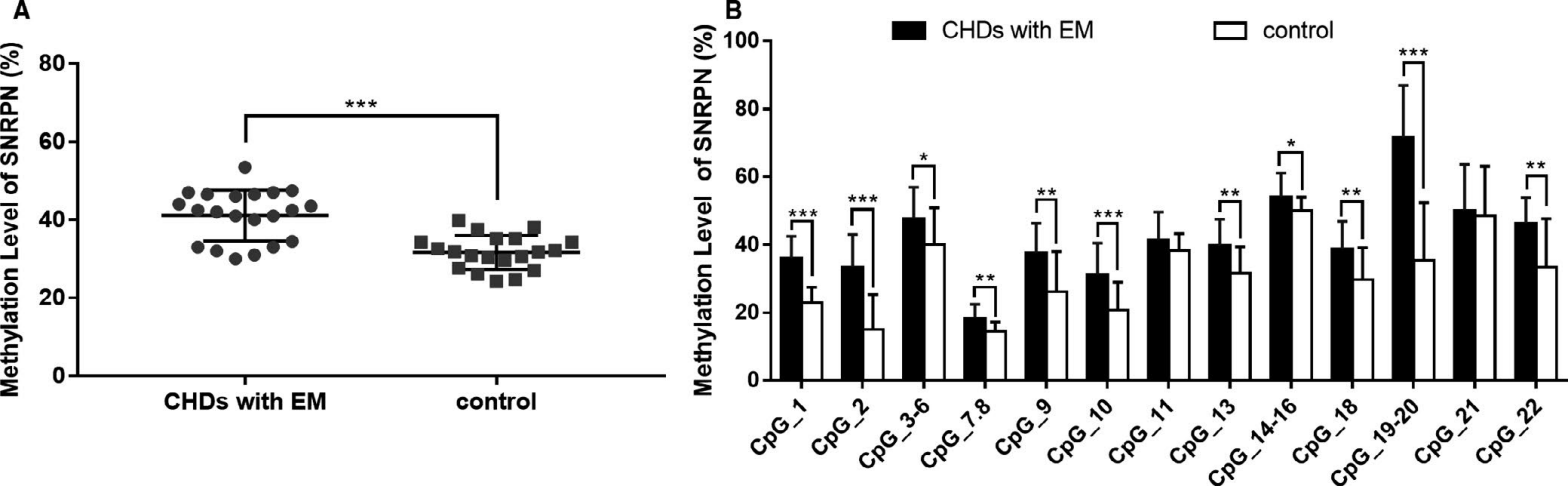
The methylation level of the *SNRPN* gene in the case group is significantly higher than that in the control group. A) Average methylation levels in the case and control groups. Each point indicates the individual average methylation levels. The differences in the DNA methylation levels (mean, range) between the groups were statistically significant [0.415 (0.383‐0.448) for the case group and 0.313 (0.274‐0.352) for the control group; Student's t test]. B) Methylation levels of specific CpG sites in the *SNRPN* gene. The CpG sites are numbered 1‐22 from the 5' end to the 3' end in the promoter area of *SNRPN*. The methylation levels of CpG sites 1‐10, 13‐20, and 22 in the case group were significantly different than those in the control group. The data are expressed as means ± SD. *P < .05, ** *P* < .01, *** *P* < .001

**Figure 2:**
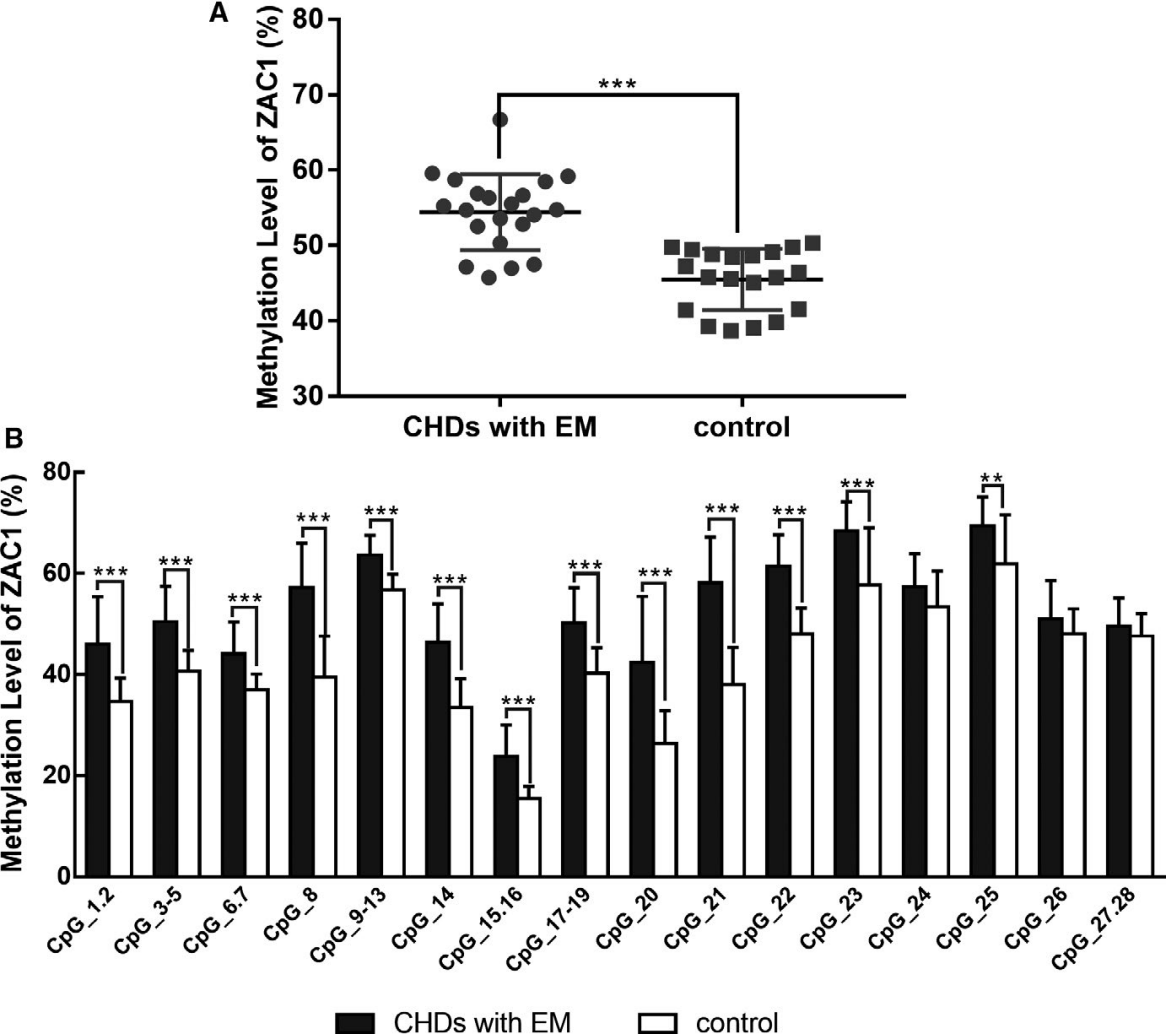
The methylation level of the *ZAC1* gene in the case group is significantly higher than that in the control group. A) Average methylation levels in the case and control groups. The boxes extend from the 25th to 75th percentiles and are divided by a line representing the median of each group. The different methylation levels between the groups of both regions were statistically significant. B) Methylation level of specific CpG sites in the *ZAC1* gene. The CpG sites are numbered 1‐28 from the 5' end to the 3' end, and the methylation levels of CpG sites numbered 1‐23 and 25 in the case group were significantly different than those in the control group. The data were expressed as means ± SD. **P < .01, ***P < .001

**Figure 3:**
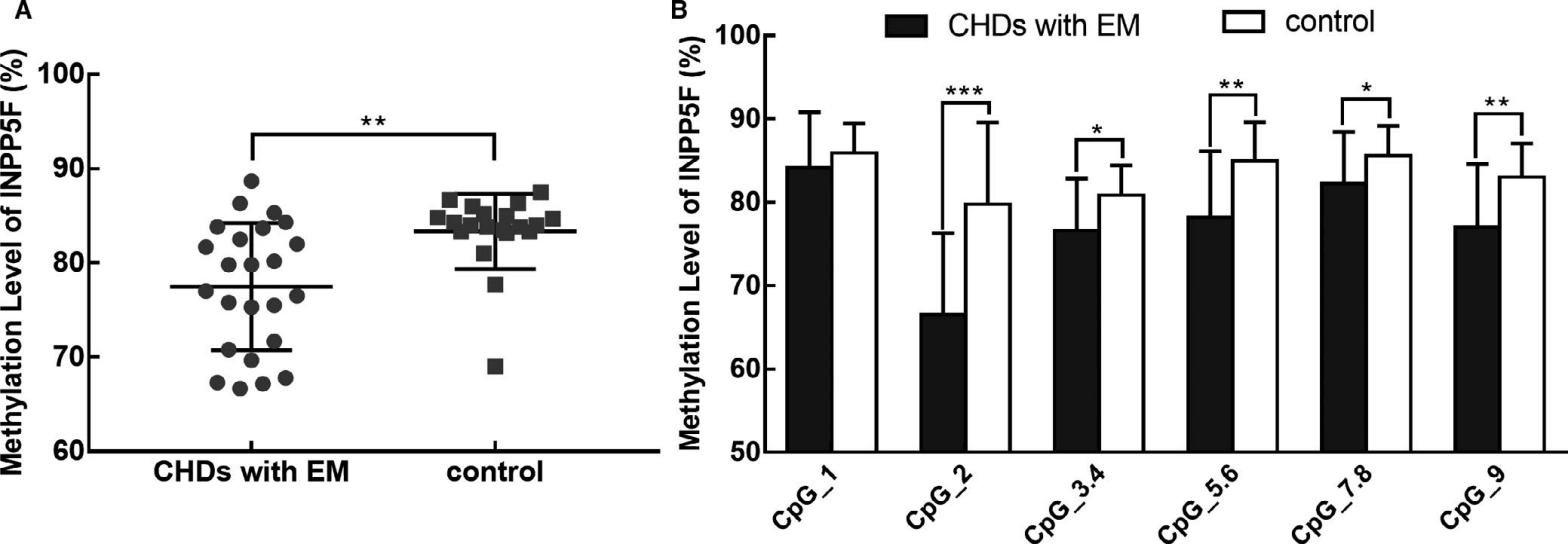
Difference between the methylation levels of the *INPP5F* gene in the case and control groups. A) Average methylation levels in cases with CHD with EM and controls. The methylation level of the case group was lower than that of the control group, and the difference was statistically significant. B) Methylation level of specific CpG sites in the *INPP5F* gene. The CpG sites are numbered 1‐9 from the 5' end to the 3' end in the promoter area of *INPP5F*. The methylation levels of CpG sites 2‐9 in the case group were significant different than those in the case group. The data were expressed as means ± SD. *P < .05, **P < .01, ***P < .001

Blood samples from 24 cases with CHD with EM and 20 control subjects were obtained for methylation analysis. The characteristics of the subjects are shown in Table 1. Three imprinted genes, *SNRPN*, *ZAC1,* and *INPP5F*, related to embryo development were selected to explore the role of imprinting modifications in CHD with EM. The methylation levels of *SNRPN*, *ZAC1,* and *INPP5F* in blood samples are shown in Table 2. Significant alterations in the methylation level were observed in these three imprinted genes in samples with multiple malformations. Compared with the control group, the methylation levels of *SNRPN* and *ZAC1* in the cases increased by approximately 10% (41.1%±6.52% vs. 31.3%±4.37%; 54.44%±5.03% vs. 45.5%±4.06%). By contrast, the methylation level of *INPP5F* was significantly decreased in cases with CHD with EM (77.48%±6.73%) compared with that in control subjects (83.36%±3.98%). Additionally, the methylation levels of each CpG site in these three imprinted genes were further evaluated, and results are separately shown in Figures 1, 2, 3. Compared with the control samples, the methylation levels of almost all CpG sites changed significantly in CHD with EM. Specifically, compared with the control samples, the sites with the largest differences in the methylation level were located on the CpG19‐20 of *SNRPN* (increased 36.4%), CpG21 of *ZAC1* (increased 20.1%) and CpG2 of *INPP5F* (decreased 13.2%). Additionally, sites with the smallest difference in methylation level included the CpG21 of *SNRPN* (increased 1.65%), CpG27‐28 of *ZAC1* (increased 1.89%), and CpG1 of *INPP5F* (decreased 1.783%).

**Table 2:** Methylation levels of the three imprinted genes in cases with CHD with EM and control subjects

Imprinted gene	Controls (Mean ± SD)	Cases (Mean ± SD)	*P* value
*SNRPN*	31.3 ±3.9%	41.1%±6.4%	<.001
*ZAC1*	42.4%±3.2%	53.9%±7.3%	<.001
*INPP5F*	83.4%±3.9%	77.5%±6.6%	<.01

### 3.2 Assessment of the risk of developing CHD with EM

We developed a model to assess the risk of developing CHD with EM based on the methylation levels of imprinted genes *SNRPN*, *ZAC1,* and *INPP5F*. CHD with EM samples were categorized according to the quartile of methylation level found in the control samples. Based on the *SNRPN* and *ZAC1* methylation levels, 70.83% and 75% of case samples were grouped into the highest quartile (methylation level ≥ 34.96% and 49.03%), respectively. By contrast, based on the *INPP5F* methylation levels, 75% of CHD with EM samples were grouped into the lowest quartile (methylation level ≤ 83.33%), and six CHD with EM samples were grouped into the highest quartile (methylation level ≥ 85.13%). High *SNRPN* and *ZAC1* methylation levels increased the risk of CHD with EM approximately 7‐ and 9‐fold, respectively [OR: 7.286, 95% confidence interval (CI): 1.905, 27.861; OR: 9.00, 95% CI: 2.286, 35.433] compared with low methylation levels (Table 3). Additionally, lower methylation levels of INPP5F statistically conferred a higher risk of CHD with EM (OR: 12; 95% CI: 2.862, 50.306). Because the methylation patterns change throughout embryonic development, we analysed the relationship between the methylation level and age and sex of the samples. After adjusting the OR to consider age and sex, the risk of CHD with EM associated with high *SNRPN* and *ZAC1* methylation levels increased [Adjusted OR (AOR): 5.454, 95% CI: 1.155, 26.622; AOR: 7.438, 95% CI: 1.531, 36.138]. Similarly, lower methylation levels of INPP5F conferred a higher risk of CHD with EM (AOR: 8.38, 95% CI: 1.712, 41.021).

**Table 3:** Risk of CHD with EM associated with the methylation levels of Imprinted genes^a^

Imprinted Gene	Methylation level	Cases n (%)	Controls n (%)	OR (95% CI)	Adjust OR^b^ (95% CI)	*P* value
*SNRPN*	Q1‐Q3	7 (29.16)	15 (75)			
	Q4	17 (70.83)	5 (25)	7.286 (1.905‐27.861)	5.545 (1.155‐26.622)	.006
*ZAC1*	Q1‐Q3	6 (25)	15 (75)			
	Q4	18 (75)	5 (25)	9 (2.286‐35.433)	7.438 (1.531‐36.138)	.002
*INPP5F*	Q1	6 (25)	16 (80)			
	Q2‐Q4	18 (75)	4 (20)	12 (2.862‐50.306)	8.38 (1.712‐41.021)	.001

^a^ Cutoffs defined as the 25th and 75th percentiles (P25 and P75, respectively) of the methylation level of the control group. OR, odds ratio.

^b^ OR adjusted by sex and age by logistic regression.

### 3.3 Expression Levels of *SNRPN*, *ZAC1,* and *INPP5F* in the blood samples of cases CHD with EM

**Figure 4:**
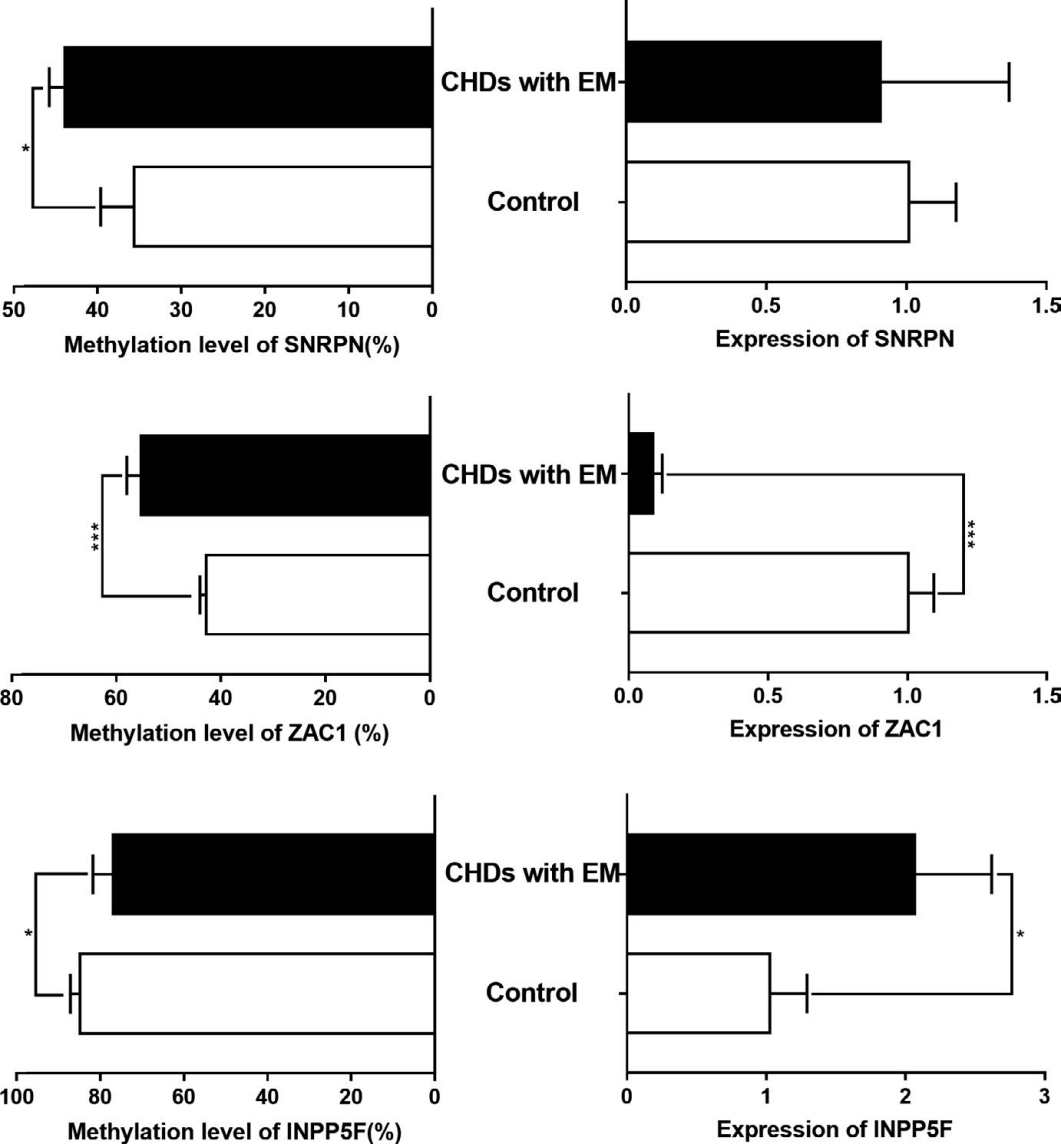
Altered expression level of *SNRPN*, *ZAC1,* and *INPP5F* accompanied by methylation level changes in cases with CHD with EM. The expression levels were compared between the case and control groups. Significantly increased expression of *ZAC1* and decreased expression of *INPP5F* were detected in the blood samples from cases with CHD with EM. The expression level of *SNRPN* showed a decreased tendency, but the difference was not statistically significant. * *P* < .05, *** *P* < .001

To determine whether the abnormal methylation of *SNRPN*, *ZAC1,* and *INPP5F* affected the expression level, we detected the expression of imprinted genes in CHD with EM patients by real‐time PCR. Compared with the control group, the expression levels of *SNRPN* and *ZAC1* in the CHD with EM group decreased 0.831 and 0.083 times, respectively, while the expression levels of *INPP5F* increased significantly two times (Figure 4).

### 3.4 *SNRPN*, *ZAC1* and *INPP5F* transcription negatively correlates with methylation modifications

**Figure 5:**
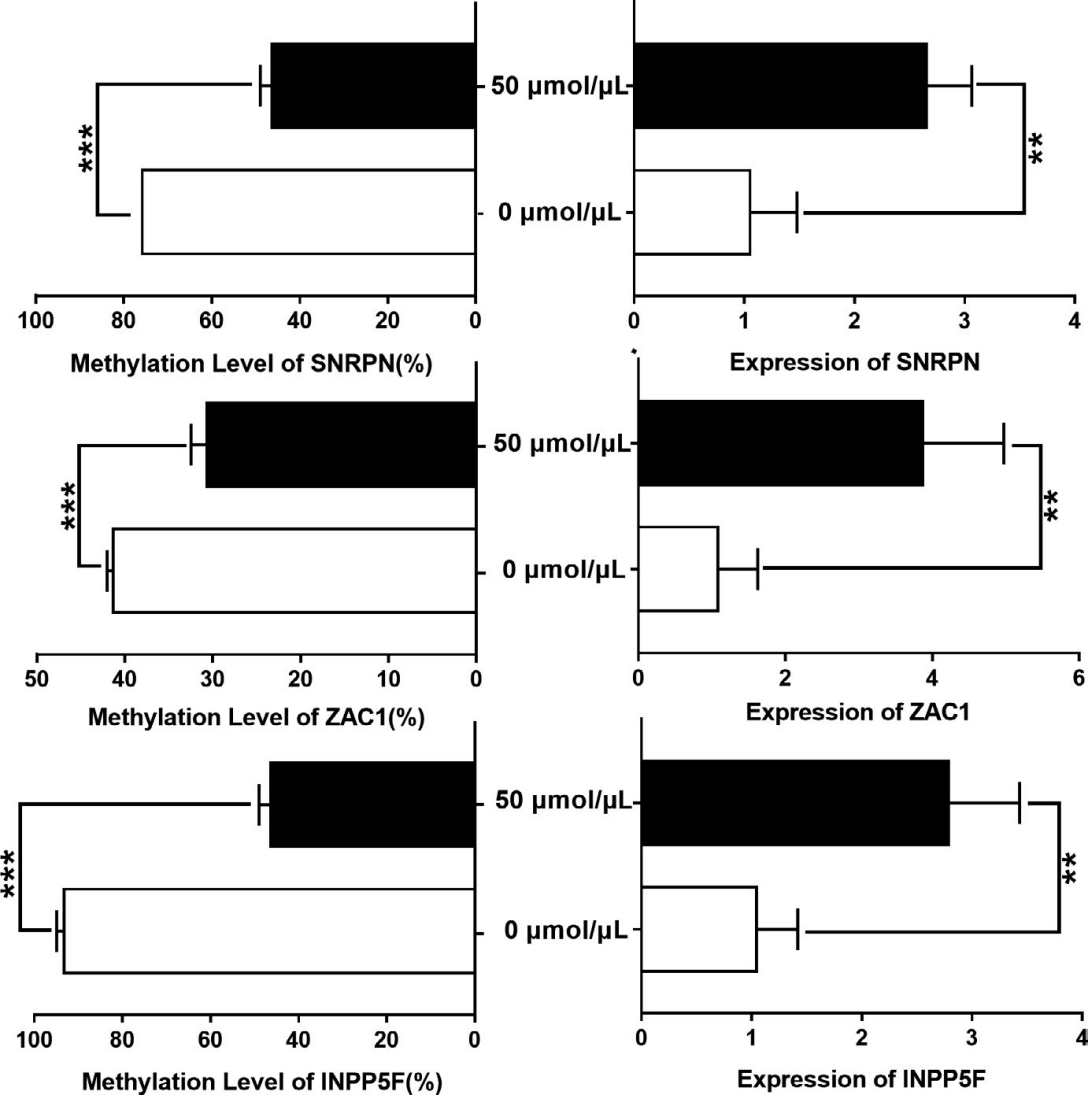
Correlation between the *SNRPN*, *ZAC1,* and *INPP5F* expression levels and methylation levels in HCT15 cells. Increased expression levels of the three imprinted genes are accompanied by hypomethylation in HCT15 cells treated with 5‐Aza. Left: The *SNRPN*, *ZAC1,* and *INPP5F* methylation levels were compared between controls (cells treated with 0 μmol/μl 5‐Aza) and cells treated with 50 μmol/μL of 5‐Aza. The methylation levels of all the genes were decreased in 50‐μmol/μL 5‐Aza group. Right: The *SNRPN*, *ZAC1,* and *INPP5F* expression levels were compared between controls and cells treated with 50 μmol/μL of 5‐Aza. The expression levels of all the genes were increased in the 50‐μmol/μL 5‐Aza group. * *P* < .05, ** *P* < .01, *** *P* < .001

To explore whether the transcription of *SNRPN*, *ZAC1,* and *INPP5F* was affected by their methylation changes, we established a methylation cell model of HCT15 treated with 5‐Aza and examined the methylation and expression levels of *SNRPN*, *ZAC1,* and *INPP5F*. HCT15 cells were treated with 5‐Aza at 0 and 50 μmol/μl (Figure 5). The methylation levels of *SNRPN*, *ZAC1* and *INPP5F* after 5‐Aza treatment were significantly reduced (93.2 ± 1.4% vs 46.3 ± 2.2%; 39.1 ± 0.4% vs 28.7 ± 0.5%; 75.7 ± 0.7% vs 46.3 ± 2.1%). Similarly, compared with the untreated group, the transcriptional expression of *SNRPN*, *ZAC1*, and *INPP5F* in the treated group increased significantly (3.78, 2.74, and 2.63 times, respectively), suggesting that the transcription levels of *SNRPN*, *ZAC1*, and *INPP5F* are sensitive to changes in DNA methylation (Figure 5). Thus, we propose the preliminary view that the transcription levels of *SNRPN*, *ZAC1*, and *INPP5F* are negatively correlated with their methylation levels, suggesting that high methylation of *SNRPN* and *ZAC1* results in transcriptional inhibition; however, *INPP5F* was hypomethylated to activate transcription (Figure 5).

## 4 DISCUSSION

The pathogenesis of CHD with EM is very complex, and the underlying mechanism remains unclear.^1^ In the present study, we demonstrated that the changed methylation modifications of imprinted genes could lead to CHD with the abnormality of different systems. Our data showed that the methylation levels of *SNRPN* and *ZAC1* increased significantly in CHD with EM, while those of *INPP5F* decreased significantly. In addition to the changed methylation modifications of these selected imprinted genes, the gene expression levels were negatively altered in CHD with EM. Interestingly, abnormal imprinting increased the risk of CHD with EM.

Imprinting is an epigenetic marking of genomes based on the parental origin, which can generate differential expression of paternal and maternal alleles in certain tissues and developmental phases. These epigenetic marks are set up in the germline and can be maintained and passed down to offspring through mitotic divisions. Changes in methylation modification can alter the traditional genetic balance of parents, finally affecting the development process of embryos and manifesting as multi‐system dysplasia, including developmental disorders of the cardiovascular system.^7^, ^8^ In this study, first of all, any samples was excluded with abnormal gene duplication/deletion after the analysis of CNVs, which means that epigenetics may contribute more to the pathogenesis of CHD with EM. On the basis, we selected gDMRs of the imprinted genes *SNRPN*, *ZAC1,* and *INPP5F* to study methylation regulation in CHD with EM. Our data are the first to hint that not only chromatin mutation but also epigenetic regulation is involved in the pathology of CHD with EM. Imprinting control regions (ICRs) of imprinted genes acquire specific imprint marks that are inherited from the male or female gamete (gDMRs). Once acquired in the germ cells, DNA methylation imprints are maintained in all the somatic lineages throughout development and function as indicators to establish imprinting modifications of the whole gene.^20^, ^21^ Some of the gDMRs are cis‐acting regulatory regions known to control the expression of more than one linked gene, further affecting the development of the whole embryo.^22^ Thus, abnormal imprinting of gDMRs may have widespread consequences on an individual's health later in life, starting from the zygote stage, affecting different organs and systems, and leading to multiple malformations.

Methylation modification is a protective form of chromosome stability; abnormal methylation could lead to a ‘metastable’ status of these genomes, resulting in chromosomal deletion and abnormal replication.^12^ Methylations occurring throughout the mitosis period affect kinetochore assembly, chromosome condensation, and segregation, which play key roles in the stability of the genome structure.^13^ Thus, abnormal methylation of imprinted genes can lead to congenital abnormalities in multiple systems.

Methylation modifications in gDMRs also affect the expression outcomes of these three imprinted genes. In cases with CHD with EM, we found a striking gain of imprinting in the *SNRPN* and *ZAC1* genes, and a loss of imprinting in the *INPP5F* gene, accompanied by gene suppression or gene activation. These findings support the hypothesis that abnormal methylation modification impairs the function of imprinted genes and contribute to the aetiology of CHD with EM. Cell models provide better supporting evidence for the correlation between the methylation modification of gDMRs and expression levels of imprinted genes. Our data suggest that abnormal gDMRs methylation levels of the imprinted genes *SNRPN*, *ZAC1,* and *INPP5F* may negatively regulate their transcriptional levels, affecting the specific functions of their proteins and downstream regulatory networks, eventually contributing to the occurrence of CHD with EM.

The *SNRPN*, *ZAC1,* and *INPP5F* genes play essential roles in embryogenesis. *SNRPN*, a member of the SMB/SMN family, participates in pre‐mRNA processing and coding through tissue‐specific variable splicing events.^23^ The gene may play an important role in specific tissues and organ development, especially the heart and brain.^23^, ^24^, ^25^ Jing et al 2015 have reported that *SNRPN* can significantly change the cell cycle distribution.^26^ Previous studies have also shown that *SNRPN* abnormal imprinting may promote the occurrence of many cancers.^27^, ^28^ *ZAC1* is a core member of the regulatory Imprinted Gene Network (IGN) and participates in activating other essential imprinted genes that control embryo growth, including *Igf2*, *H19*, *Cdkn1c*, and *Dlk1*.^29^, ^30^ *ZAC1* is an important transcription factor that is mainly expressed in the new moon phase of heart development and influences heart formation.^31^ *ZAC1* can form complexes with *Nkx2‐5* and the *ANF/Nppa* gene and affect heart development by synergistically activating the expression of related genes.^32^ Some studies have also reported that the hypermethylation of *ZAC1* can lead to changes in the IGN, causing embryonic growth restriction, neonatal diabetes, and other diseases.^29^, ^33^ The imprinted gene *INPP5F* is highly expressed in the heart, brain, and other tissues. Some studies have shown that *INPP5F* is an essential endogenous regulator of myocardial cell size and the cardiac stress response and could reduce *PIP3* levels, subsequently activating Akt and downstream signalling.^34^, ^35^ The Akt signal transduction network functions to regulate cardiac adaptability (physiological) and maladjustment (pathological) hypertrophy in heart formation.^36^, ^37^ The *INPP5F*‐encoded Sac2 protein also plays a role in endocytosis.^38^

In our study, hypermethylation of *SNRPN* of *ZAC1* and hypomethylation of *INPP5F* accompanied by abnormal gene expressions were observed in CHD with EM cases, suggesting that imprinting aberrations may be an important regulator that causes the abnormal growth and development of embryonic organs, leading to CHD with EM. However, limited by the number of enrolled cases in this study, further mechanistic exploration is needed to confirm the effect of imprinted genes in the pathology of CHD with EM.

## 5 CONCLUSION

Imprinting aberrations were first reported in the pathology of CHD with EM. Our data imply that abnormal hypermethylation of *SNRPN* and *ZAC1* and hypomethylation of *INPP5F* are involved in the increasing risk of CHD with EM by altering their gene expression. This study provides experimental data concerning the role of imprinting regulation in the pathology of multiple malformations.

## CONFLICT OF INTEREST

The authors declare that they have no conflict of interest.

## AUTHOR CONTRIBUTION

**Xiaolei Zhao:** Data curation (lead); Formal analysis (lead); Investigation (lead); Resources (lead); Writing‐original draft (equal); Writing‐review & editing (equal). **Shaoyan Chang:** Writing‐original draft (equal); Writing‐review & editing (equal). **Xinli Liu:** Data curation (equal). **Shuangxing Wang:** Data curation (equal); Resources (equal). **Yueran Zhang:** Resources (equal). **Xiaolin Lu:** Investigation (equal); Software (equal). **Ting Zhang:** Funding acquisition (equal); Project administration (equal). **Hui Zhang:** Conceptualization (equal); Data curation (equal); Funding acquisition (equal); Methodology (equal); Project administration (equal); Resources (equal). **Li Wang:** Conceptualization (equal); Funding acquisition (equal); Methodology (equal); Project administration (equal); Supervision (equal); Writing‐original draft (equal); Writing‐review & editing (equal). WL and ZH designed the study. ZXL conducted all experiments and statistical analysis. ZXL and WSX collected the blood samples. LXL and ZYR participated in the cell culture experiments. ZXL, CSY and WL finalized the manuscript. All the authors read and approved the final manuscript.

## Supporting information

Supplementary Material

## Data Availability

The data that support the findings of this study are available from the corresponding author upon reasonable request.
