# Supplementary material for: Imprinting aberrations of SNRPN, ZAC1 and INPP5F genes involved in the pathogenesis of congenital heart disease with extracardiac malformations
Source: J Cell Mol Med. 2020 Jul 21;24(17):9898–907. doi: 10.1111/jcmm.15584 (PMC7520315; doi:10.1111/jcmm.15584)
Supplement: Supplementary file 1 — Supplementary Material [file JCMM-24-9898-s001.docx]

**Supplementary 1**

| Table S1 Primer Sequences of SNRPN, ZAC1 and INPP5F for Massarray | |
| --- | --- |
| Genes | Primer |
| SNRPN-L | aggaagagagTGTGGGGTTTTAGGGGTTTAG |
| SNRPN-R | cagtaatacgactcactatagggagaaggctCTCCCCAAACTATCTCTTAAAAAAA |
| ZAC1-H1L | aggaagagagGGGTTGAATGATAAATGGTAGATG |
| ZAC1-H1R | cagtaatacgactcactatagggagaaggctACCTTAACTTTACCCCCACC |
| ZAC1-H3L | aggaagagagATTTTTTTTAGTGTTGTTGTGAGGA |
| ZAC1-H3R | cagtaatacgactcactatagggagaaggctCCAAAACCCAATCACACATAA |
| INPP5F-L | aggaagagagGAAGTAAGTTGGGAATATTATTTGG |
| INPP5F-R | cagtaatacgactcactatagggagaaggctCTATCCTAACCCTTCTCCCATACA |

| Table S2 Real-Time PCR Primers of SNRPN, ZAC1 and INPP5F | |
| --- | --- |
| Genes | Primer |
| SNRPN-L | AAGATCAAGCCAAAGAATGC |
| SNRPN-R | CATGGATACCAAGTTCTCCC |
| ZAC1-L | TTATTCCCACTCCAGGGAG |
| ZAC1-R | ATGGGTAGCCATATGCCTC |
| INPP5F-L | TTGAAATAGGCCCTGAACC |
| INPP5F-R | TCGCTTCTTTGTATCTGTAGTG |
| GAPDH-L | CTGACTTCAACAGCGACACC |
| GAPDH-R | GTGGTCCAGGGGTCTTACTC |

**Supplementary 2**

Analyzed by extracardiac malformations, we didn’t find out the significant differences in methylation levels between different extracardiac systems (Table S3). We also tried to compared the methylation levels between CHD subgroups (VSD, ASD, PDA, COA and others), which showed no significant differences also (Table S4).

| Table S3 Methylation level differences among different extracardiac systems | | | | |
| --- | --- | --- | --- | --- |
| Extracardiac Systems | | p value | | |
|  |  | SNRPN | ZAC1 | INPP5F |
| Urinary | Digestive | 0.623 | 0.271 | 0.999 |
|  | Nervous | 0.317 | 0.540 | 0.578 |
|  | Motor | 0.072 | 0.259 | 0.891 |
|  | Others | 0.955 | 0.414 | 0.411 |
| Digestive | Nervous | 0.187 | 0.766 | 0.656 |
|  | Motor | 0.062 | 0.646 | 0.909 |
|  | Others | 0.704 | 0.819 | 0.496 |
| Nervous | Motor | 0.693 | 0.369 | 0.698 |
|  | Others | 0.365 | 0.878 | 0.379 |
| Motor | Others | 0.062 | 0.346 | 0.712 |

| Table S4 Methylation level differences among different CHD subgroups | | | | |
| --- | --- | --- | --- | --- |
| CHD subgroups | | p value | | |
|  |  | SNRPN | ZAC1 | INPP5F |
| VSD | ASD | 0.946 | 0.709 | 0.849 |
|  | PDA | 0.583 | 0.830 | 0.182 |
|  | COA | 0.434 | 0.453 | 0.260 |
|  | Others | 0.445 | 0.707 | 0.050 |
| ASD | PDA | 0.595 | 0.608 | 0.116 |
|  | COA | 0.463 | 0.562 | 0.177 |
|  | Others | 0.562 | 0.456 | 0.025 |
| PDA | COA | 0.892 | 0.385 | 0872 |
|  | Others | 0.364 | 0.909 | 0.812 |
| COA | Others | 0.269 | 0.245 | 0.646 |
